# Restoration Approaches Regulate Soil Multifunctionality via Microbial Nitrogen and Plant Diversity in Northern Forests

**DOI:** 10.1002/ece3.73642

**Published:** 2026-05-19

**Authors:** Yonghong Luo, Ye Liu, Peng Wang, Jiazhi Wang, Fuji Liu, Shuhua Wei, Xingfu Yan, Jiming Cheng

**Affiliations:** ^1^ School of Biological Science and Engineering North Minzu University Yinchuan China; ^2^ School of Ecology and Environment Inner Mongolia University Hohhot China; ^3^ College of Geography and Planning Ningxia University Yinchuan China; ^4^ Chengde Meteorological Disaster Prevention Center of Hebei Province Chengde Hebei China; ^5^ Ningxia Academy of Agriculture and Forestry Sciences Plant Protection Institute Yinchuan China; ^6^ Key Laboratory of Ecological Protection of Agro‐Pastoral Ecotones in the Yellow River Basin National Ethnic Affairs Commission of the People's Republic of China Yinchuan China

**Keywords:** artificial restoration, natural restoration, plant diversity, *Quercus wutaishanica*, soil microbial biomass

## Abstract

Long‐term anthropogenic disturbances have damaged forest ecosystems, and different restoration approaches after disturbance play a crucial regulatory role in maintaining and enhancing ecosystem functions. However, existing studies have mostly focused on the response characteristics of single ecosystem functions, while research on the impacts of different restoration methods on soil multifunctionality of forests and their driving mechanisms remains scarce. Taking the completely anthropogenically disturbed *Quercus wutaishanica* communities in northern deciduous broad‐leaved forests as the research object, this study systematically explored the comprehensive effects of two typical restoration approaches (natural restoration and artificial assisted restoration with *Larix principis‐rupprechtii*) and soil layer depths (0–10 cm, 10–20 cm) on soil microbial biomass and soil multifunctionality after 40 years of restoration, and further revealed the dominant driving factors of soil multifunctionality. The results showed that: (1) Artificial assisted restoration markedly increased soil microbial phosphorus content by 97.52% ± 28.86%; with increasing soil depth, both soil microbial carbon and nitrogen contents decreased significantly, by 24.18% ± 6.14% and 7.89% ± 2.12%, respectively. (2) Artificial assisted restoration communities exhibited a significantly higher soil multifunctionality index (0.34) compared with natural restoration communities (−0.37). Further analysis indicated that soil multifunctionality was mainly driven by soil microbial nitrogen content and the Pielou evenness index of plant community composition. This study provides notable theoretical supports and practical references for the sustenance, renewal and management of damaged ecosystems in northern deciduous broad‐leaved forests.

## Graphical abstract

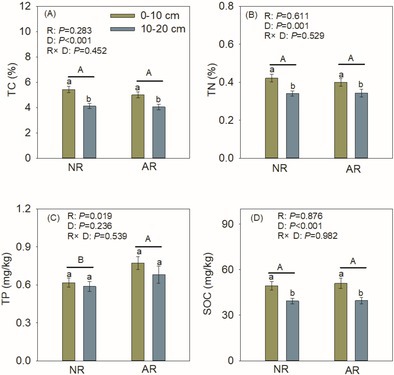

## 1 Introduction

Forests are among Earth's most critical ecosystems, providing habitats for numerous organisms and sustaining ecological balance, regional climates, and soil conservation (Kumar et al. 2022). However, intensified anthropogenic activities (e.g., large‐scale grazing, unregulated logging) have severely perturbed forest ecosystems in recent decades, damaging biodiversity and functionality (Wang et al. 2025; Yu et al. 2022). To mitigate degradation, various restoration strategies have been implemented (Aronson et al. 2020), with soil playing a pivotal role in mediating restoration success (Moreno‐Mateos et al. 2020). Thus, investigating how different restoration approaches affect belowground microbial activities is imperative.

Soil microbes are fundamental to key soil processes (e.g., organic matter decomposition, nutrient mobilization) and underpin soil multifunctionality (Pedrinho et al. 2024; Smith et al. 2015). Microbial biomass, a key indicator of soil nutrient cycling and health, is closely linked to soil physicochemical properties, plant diversity, and soil depth (Čapek et al. 2021; Liu et al. 2018; Thakur et al. 2015). These factors vary with restoration approaches (Rosenzweig et al. 2016; Wei et al. 2023), potentially altering microbial biomass dynamics.

Soil multifunctionality integrates multiple biogeochemical and physical indicators to robustly quantify soil ecosystem responses to disturbances (Long et al. 2025; Zwetsloot et al. 2021) and is widely used to assess restoration (Guo et al. 2021; Liu et al. 2025). While prior studies focus on single ecosystem functions, the effects of different restoration approaches on forest soil multifunctionality post‐disturbance remain unclear; notably, soil multifunctionality is jointly driven by biotic and abiotic factors, with microbial biomass and plant diversity as core regulators (Shen et al. 2024; Delgado‐Baquerizo et al. 2017), yet research on its drivers has largely focused on microbial assemblages, leaving the roles of plant diversity and microbial biomass post‐disturbance poorly understood and thus hindering the development of effective restoration strategies.

*Quercus wutaishanica* is a dominant species in northern China's temperate forests, critical for soil conservation and ecosystem services (Cheng et al. 2024). Anthropogenic disturbances since the 1980s have degraded these forests, prompting natural restoration and artificial restoration via Larix principis‐rupprechtii planting (Luo et al. 2025). Based on the aforementioned literature, this study proposes two hypotheses: (1) Given that soil physicochemical properties and plant diversity vary with restoration approaches (Rosenzweig et al. 2016; Wei et al. 2023) and both factors regulate soil multifunctionality (Delgado‐Baquerizo et al. 2017; Xu et al. 2025), we hypothesize that there are significant differences in soil ecosystem multifunctionality between the two restoration approaches. (2) Since microbial biomass and plant diversity are core regulators of soil multifunctionality (Shen et al. 2024; Delgado‐Baquerizo et al. 2017) and are closely associated with restoration‐induced changes (Thakur et al. 2015; Wei et al. 2023), we hypothesize that plant diversity and soil microbial biomass are the main drivers of soil ecosystem multifunctionality in the study area.

## 2 Materials and Methods

### 2.1 Study Site

**FIGURE 1:**
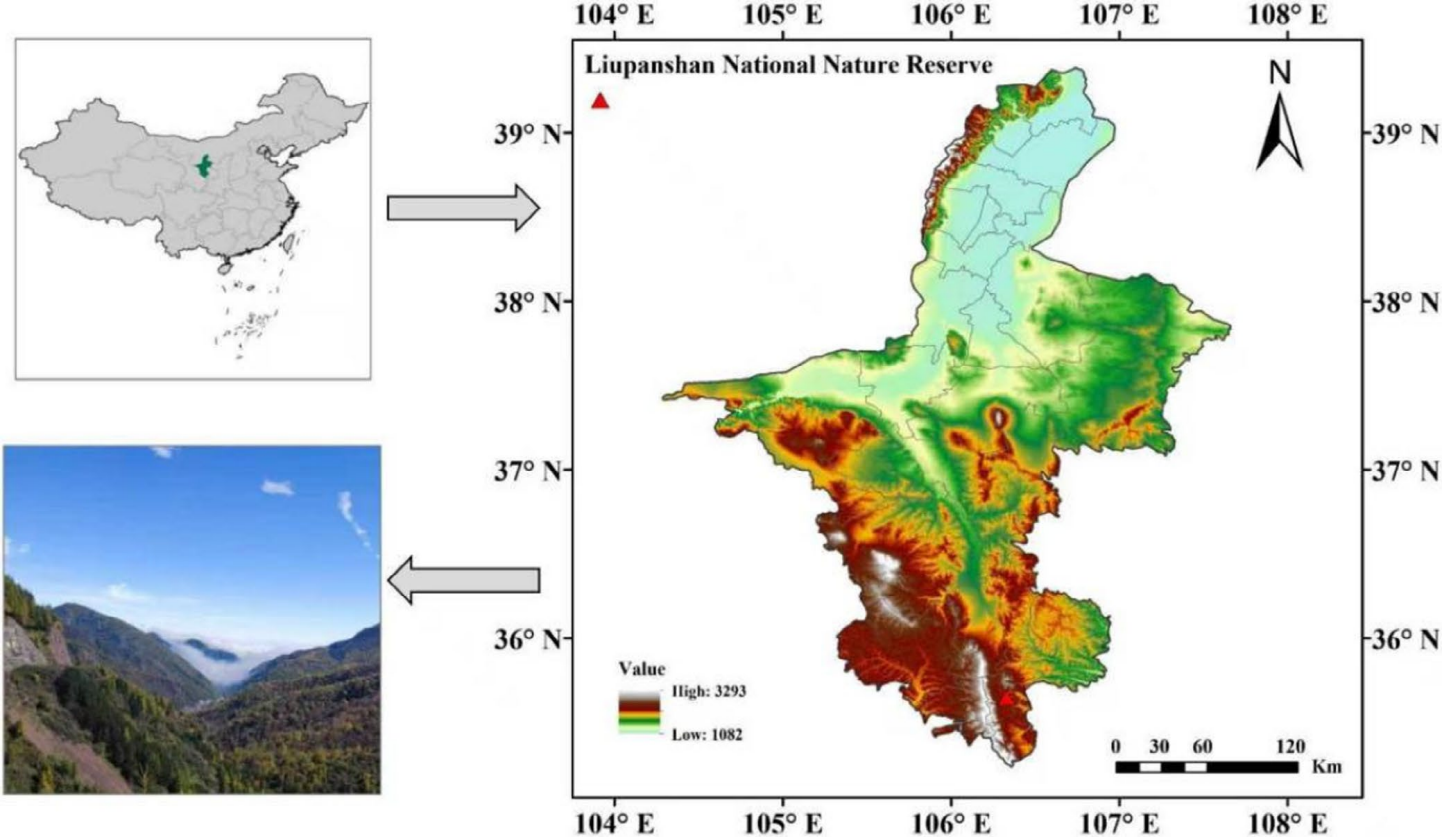
Geographical location of the study area.

The core study area was set in the Liupan Mountain National Nature Reserve (35°15′–35°41′ N, 106°09′–106°30′ E) (Figure 1), which is located at the ecologically sensitive edge of the agro‐pastoral transition zone in northern China. The climate in the study area exhibits significant seasonal variations, with an average annual temperature of 5.8°C. The average temperatures in January and July are −7°C and 17.4°C, respectively. In terms of the hydrological environment, the annual precipitation is 676 mm with a distinct seasonal pattern, and more than 70% of the precipitation occurs from June to September. The annual evaporation capacity reaches 1426 mm, far exceeding the precipitation, forming a semi‐arid hydrological pattern in the study area (Cheng et al. 2024). Soils in the study area, classified according to the Chinese Soil Taxonomy, are dominated by Cambosols with occasional Argosols, Isohumosols and other soil types, where cool and humid Cambosols corresponding to traditional gray‐cinnamon soils dominate high‐altitude forest areas, Isohumosols occur in alpine meadows, weakly developed Orthic Cambosols are mainly distributed on steep slopes and in gullies, and gray‐cinnamon soils as the main zonal soil account for more than 90% of the forest soil area (Liu et al. 2021).

Historically, the primeval forests in this region have been severely damaged due to deforestation and overgrazing (Cheng et al. 2024). To restore the damaged vegetation, the Liupan Mountain Forestry Bureau implemented closed forestation measures in the early 1980s, creating basic conditions for vegetation restoration by delineating closed areas and prohibiting logging and grazing; at the same time, to accelerate the ecological restoration process, artificial planting of Larix principis‐rupprechtii was carried out in the region. Currently, two typical communities have been formed in the study area—naturally restored secondary forest communities and artificially planted Larix principis‐rupprechtii communities—providing ideal research objects for comparative analysis of the ecological effects of different restoration models.

In August 2025, after consulting the staff of the Liupan Mountain Forestry Bureau, two types of communities with a restoration period of approximately 40 years were selected for research in Longtan Forest Farm (35°23′ N, 106°21′ E), the core area of the reserve. Their specific characteristics are as follows: The first is a naturally restored plant community, dominated by *Q. wutaishanica*. The community structure is clear: the arbor layer mainly includes *Q. wutaishanica, Tilia paucicostata*, and other tree species; the shrub layer is dominated by *Crataegus kansuensis* , *Cotoneaster multiflorus* , *Eleutherococcus senticosus* , and other species; the herb layer is mainly distributed with *Epipactis helleborine* , *Carex tristachya*, *Thalictrum aquilegiifolium* , and other plants. The second is an artificially assisted restored *L. principis* *‐rupprechtii* community, whose dominant species include *Larix principis‐rupprechtii*, *Q. wutaishanica*, and *Tilia paucicostata*. The species composition of each layer in this community is clear: the arbor layer takes *L. principis‐rupprechtii* as the core, accompanied by *Q. wutaishanica*, *Tilia paucicostata*, and other tree species; the shrub layer mainly includes *Crataegus kansuensis* , *Rosa xanthina* , *Cotoneaster multiflorus* , and so on; the herb layer is mainly composed of *Clematis florida* , *Neillia sinensis*, *Stellaria patens*, and other species.

### 2.2 Vegetation Survey

To explore species diversity and community structure under two restoration approaches, three transects were selected for *Q. wutaishanica* and *L. principis‐rupprechtii* in the Dadaogou plot. Three sampling lines were placed at lower, middle, and upper slopes of each transect (intervals ≥ 20 m), with one random 10 m × 10 m arbor quadrat per line (18 quadrats total; 9 per method; 100 m^2^ per quadrat). All woody plants (basal diameter ≥ 1 cm) were surveyed, with data recorded on species identity, height, abundance, DBH, and crown width. The plant species diversity index was calculated as follows:

(1) Species richness:S=Total species

(2) $$\text{Shannon}–\text{Wiener index}:H^{'}=-\sum \left(p_{i}\times \log_{2}\;p_{i}\right)$$

where p is the relative abundance of species *i* in the plant community.

(3) $$\text{Simpson diversity index}:D=-\sum p_{i}^{2}$$

where p is the relative abundance of species *i* in the plant community.

(4) $$\text{Pielou evenness index}:J=H^{'}/\log_{2}\left(S\right)$$

where $H^{'}$ is the Shannon–Wiener index, and S is the number of species.

### 2.3 Soil Sampling

Soil sampling was carried out in mid‐August 2025, corresponding to the main growing season of plants in the study area. Three 5 m × 5 m quadrats were randomly positioned at lower, middle, and upper slopes in plots of naturally and artificially restored communities. Three 1.5 m × 1.5 m sub‐quadrats were established in a zig‐zag pattern within each quadrat. Soil samples from two layers (0–10 cm and 10–20 cm), which are the most biologically active and sensitive to restoration, were collected at random diagonal positions using a soil auger. Samples of the same depth from the three sub‐quadrats were mixed, sealed, and transported to the laboratory. A total of 36 soil samples were collected, following the design: 2 restoration approaches × 3 plots × 3 quadrats × 2 soil depths.

Soil water content (constant weight method; Luo et al. 2024), electrical conductivity (EC) (soil‐water ratio dilution at a soil‐to‐water ratio of 1:5; Nadler 1982), pH (soil‐water dilution potentiometry at a soil‐to‐water ratio of 1:2.5; Miller and Kissel 2010), organic carbon (potassium dichromate heating; Walinga et al. 1992), total nitrogen (Kjeldahl; Bremner and Mulvaney 1982), total/available phosphorus (molybdenum‐antimony anti‐colorimetric; Kowalenko and Babuin 2007), and ammonium/nitrate nitrogen (ultraviolet spectrophotometry; Cawse 1967) were determined using standard protocols. Microbial biomass C, N, P (MBC, MBN, MBP) were quantified via chloroform fumigation‐extraction (Brookes et al. 1985; Jeannotte et al. 2004; Vance et al. 1987). Soil β‐glucosidase (BG), N‐acetyl‐β‐glucosidase, and acid phosphatase (AP) activities were assayed by microplate fluorometry (Marx et al. 2001).

In this study, indicators related to soil carbon (total carbon, organic carbon, BG), nitrogen (total nitrogen, ammonium nitrogen, nitrate nitrogen, and N‐acetyl‐β‐glucosidase), and phosphorus (total phosphorus, available phosphorus, AP) nutrient cycling were selected as the evaluation indicators for soil ecosystem multifunctionality. These indicators also serve as important evaluation criteria for ecological function restoration (Qiu et al. 2018). Soil multifunctionality was calculated using the mean of “Z‐scores” method, with the specific calculation formula as follows (Bowker et al. 2013):

(5) $${\mathrm{MF}}_{a}=g\left(r_{i}\left(f_{i}\right)\right)/F$$

${\mathrm{MF}}_{a}$ represents ecosystem multifunctionality, $f_{i}$ represents the measured value of function i, $r_{i}$ is the mathematical function that converts $f_{i}$ into a positive value, g represents the normalization of all measured values, and F represents the number of functions measured.

### 2.4 Statistical Analysis

To systematically address the research objectives, a hierarchical statistical analysis framework was employed, with all analyses conducted in R software (version 4.3.1). The analytical process was structured to first quantify the effects of core explanatory variables (restoration method and soil depth) on key response variables, then verify group differences, and finally clarify the interactive relationships among variables and their contributions to soil multifunctionality, as detailed below:

First, linear mixed‐effects models (LMMs) were constructed to examine the effects of restoration method (natural vs. artificial) and soil depth (0–10 cm vs. 10–20 cm) on soil multifunctionality, soil physicochemical properties, microbial biomass, and enzyme activities. In these models, restoration method and soil depth were set as fixed effects to test their independent and interactive impacts, while plot was included as a random effect to account for within‐plot non‐independence (i.e., spatial autocorrelation among samples from the same plot). Separately, an additional LMM was established to specifically evaluate the effect of restoration method on plant diversity, where restoration method served as the fixed factor and plot as the random factor. All LMMs were implemented using the lmer() function in the lme4 package (version 1.1‐34).

Following model validation (to ensure compliance with assumptions of normality, homoscedasticity, and independence of residuals), post hoc analyses were performed to identify specific group differences. Tukey's honest significant difference tests (via the emmeans package [version 1.8.9]) were applied for pairwise comparisons among restoration method treatments; significant differences (*p* < 0.05) were denoted by uppercase letters in the corresponding figures. For comparisons between soil depths (0–10 cm vs. 10–20 cm), paired *t*‐tests were conducted, with significant differences (*p* < 0.05) marked by lowercase letters.

Subsequently, Pearson correlation analysis was carried out to quantify the linear relationships among soil physicochemical properties, plant diversity, and microbial biomass, with statistical significance set at *p* < 0.05. To further disentangle the relative explanatory power of biotic (e.g., plant diversity, microbial biomass) and abiotic (e.g., soil physicochemical properties) factors on soil multifunctionality, a random forest model was employed using the randomForest package (version 4.7‐1.1). Model parameters were set as follows: number of decision trees (ntree) = 500, and number of variables randomly sampled at each split (mtry) = default. To ensure model reliability, 10‐fold cross‐validation was performed to test stability. Variable importance was ranked based on the percentage increase in mean squared error (%IncMSE), and the significance of each variable's contribution was verified via permutation tests.

## 3 Result

### 3.1 The Effects of Restoration Methods and Soil Depth on Soil Multifunctionality Index and Microbial Biomass

**FIGURE 2:**
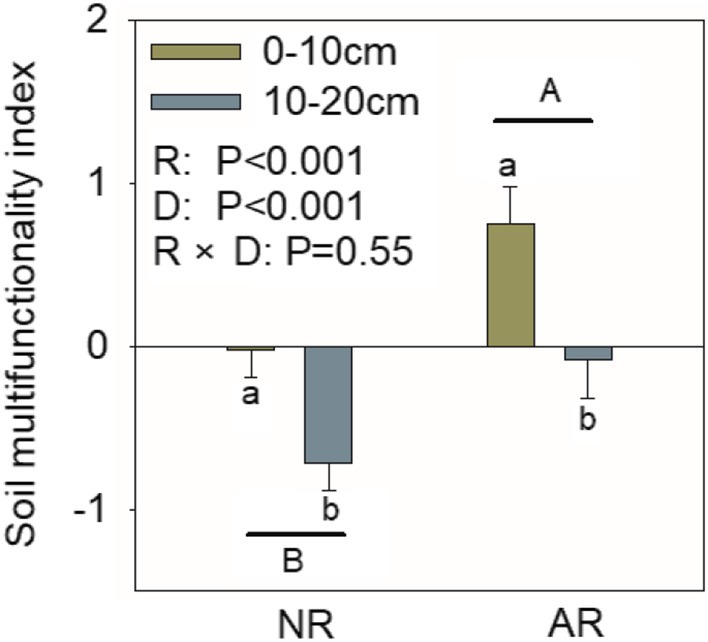
The effects of restoration methods and soil depth on the soil multifunctionality index in the Liupan Mountain area. R, D, NR, and AR denote restoration type, soil layer depth, naturally recovered community, and artificially restored community, respectively. Different uppercase letters indicate significant differences in the soil multifunctionality index among different restoration types (*p* < 0.05), while different lowercase letters indicate significant differences in the soil multifunctionality index among different soil layer depths under the same restoration type (*p* < 0.05).

**FIGURE 3:**
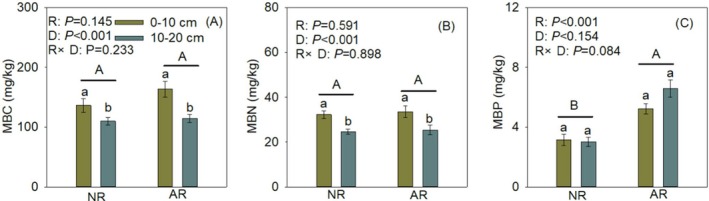
The effects of restoration methods and soil depth on the microbial biomass in the Liupan Mountain area. MBC, MBN and MBP denote microbial biomass carbon (MBC), microbial biomass nitrogen (MBN), and microbial biomass phosphorus (MBP), R, D, NR, and AR denote restoration type, soil layer depth, naturally recovered community, and artificially restored community, respectively. Different uppercase letters indicate significant differences in the soil microbial biomass different restoration types (*p* < 0.05), while different lowercase letters indicate significant differences in the soil microbial biomass among different soil layer depths under the same restoration type (*p* < 0.05).

The soil multifunctionality index of artificially assisted restored plant communities was significantly higher than that of naturally restored communities, and the soil multifunctionality index in the 0–10 cm soil layer was markedly higher than in the 10–20 cm layer under both restoration approaches (Figure 2). The soil microbial phosphorus content in artificially restored plant communities was significantly higher than in naturally restored communities, and microbial biomass carbon and nitrogen contents in the 0–10 cm soil layer were both markedly higher than in the 10–20 cm layer under both restoration approaches. (Figure 3A–C).

### 3.2 The Effect of Restoration Methods on Plant Community Diversity in the Liupan Mountain Area

**FIGURE 4:**
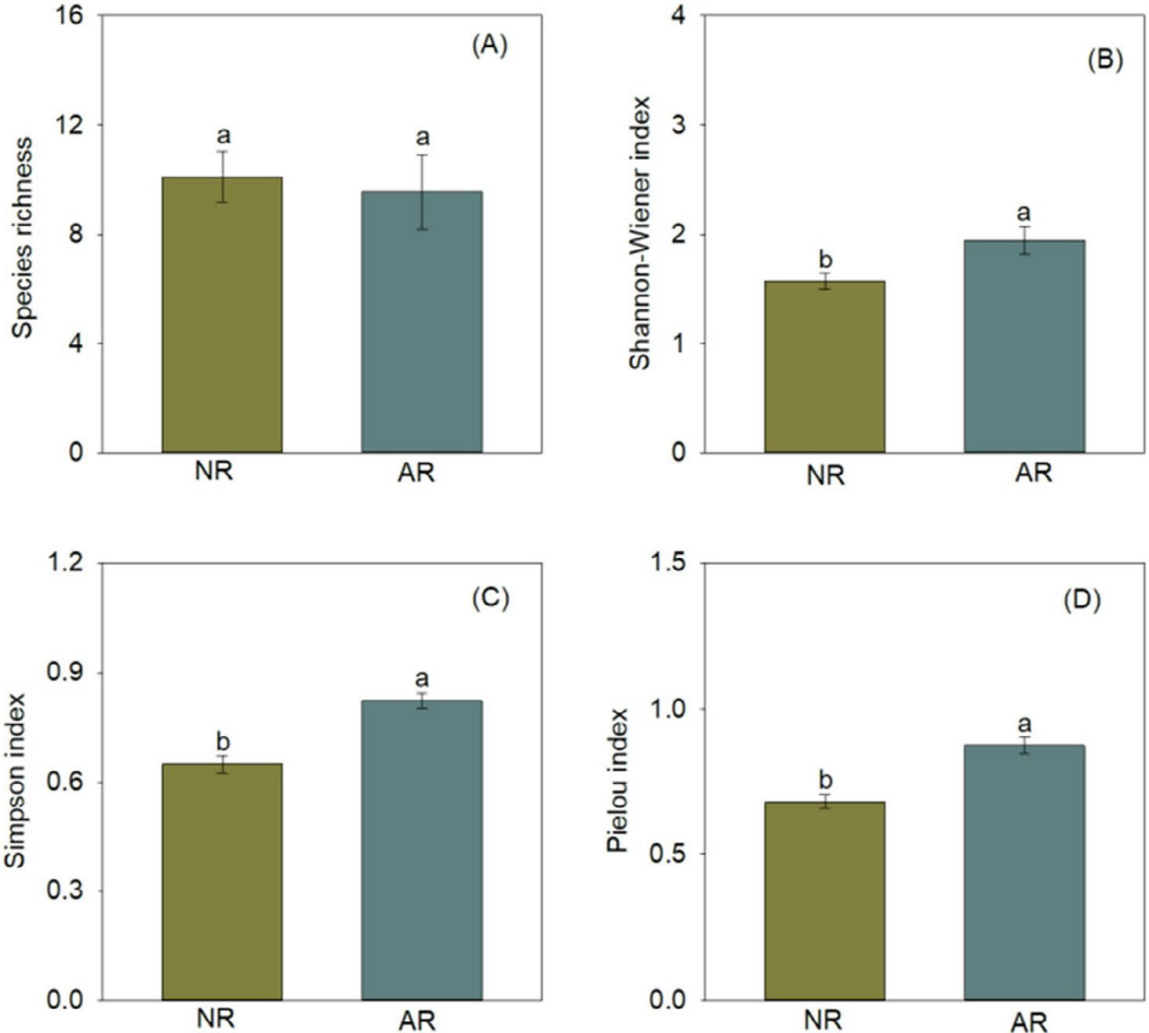
The effects of natural restoration and artificial restoration on plant diversity in the Liupan Mountain area. NR, and AR denote naturally recovered community and artificially restored community. Different lowercase letters indicate significant differences in plant diversity between the two restoration types (*p* < 0.05).

There was no significant difference in species richness between the naturally restored and artificially restored plant communities (Figure 4A). However, the Shannon–Wiener index, Simpson index, and Pielou index of the artificially restored communities were all significantly higher than those of the naturally restored communities (Figure 4B,C).

### 3.3 Responses of Soil Properties and Available Nutrients to Different Restoration Modes and Soil Layer Depths

**FIGURE 5:**
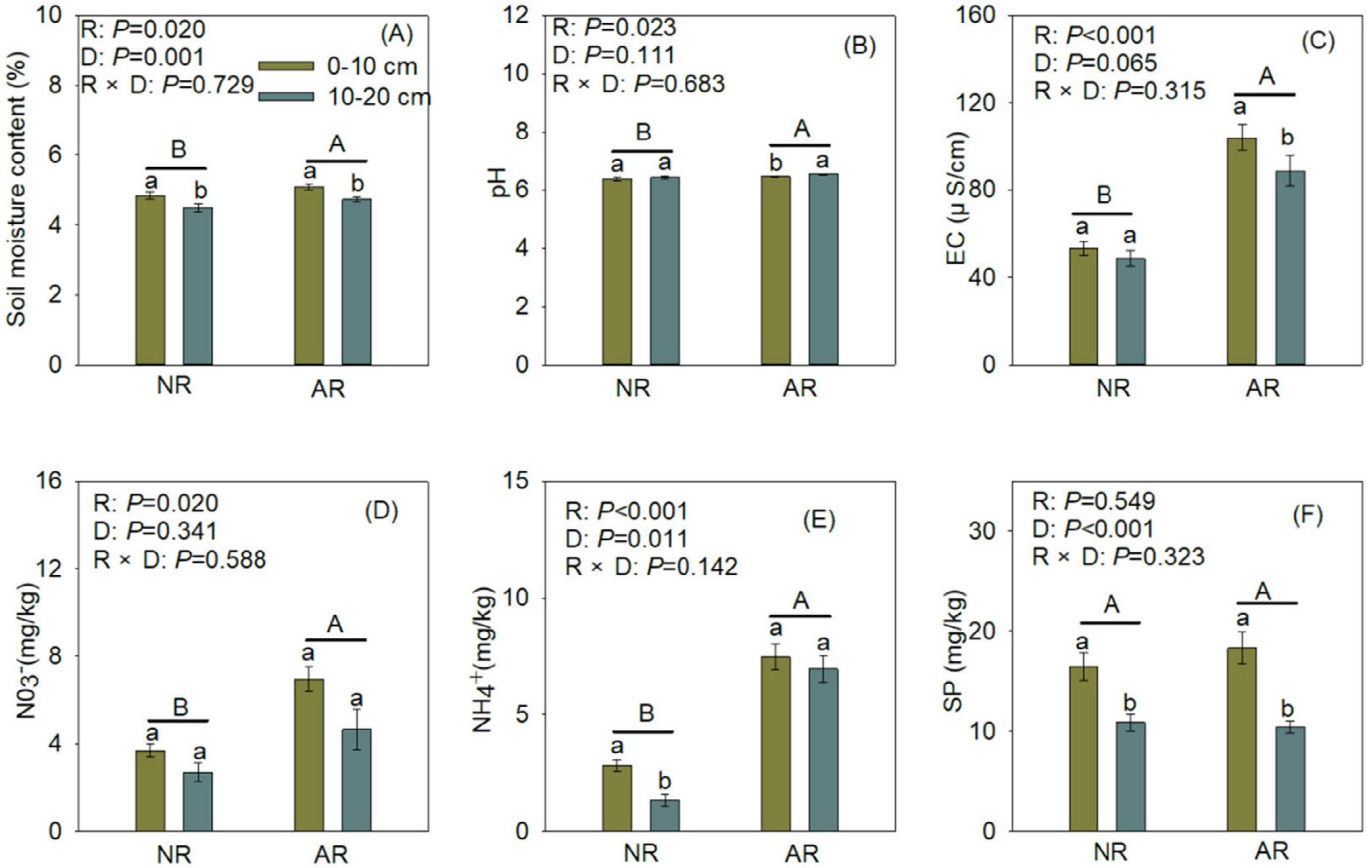
Effects of different restoration methods and soil layer depth on soil physical and chemical properties and available nutrients. NR, and AR denote naturally recovered community and artificially restored community. EC and SP represent soil electrical conductivity and soil available phosphorus respectively. Different uppercase letters indicate significant differences in the soil properties and soil available nutrients different restoration types (*p* < 0.05), while different lowercase letters indicate significant differences in the soil properties and soil available nutrients among different soil layer depths under the same restoration type (*p* < 0.05).

The soil water content, pH value, EC, nitrate nitrogen content, and ammonium nitrogen content of the artificially restored communities were significantly higher than those of the naturally restored communities (Figure 5A–E). The soil water content, ammonium nitrogen content, and soil phosphorus content in the 0–10 cm soil layer were markedly higher than those in the 10–20 cm soil layer (Figure 5A,E,F).

### 3.4 Responses of Soil Nutrients to Restoration Modes and Soil Layer Depths

**FIGURE 6:**
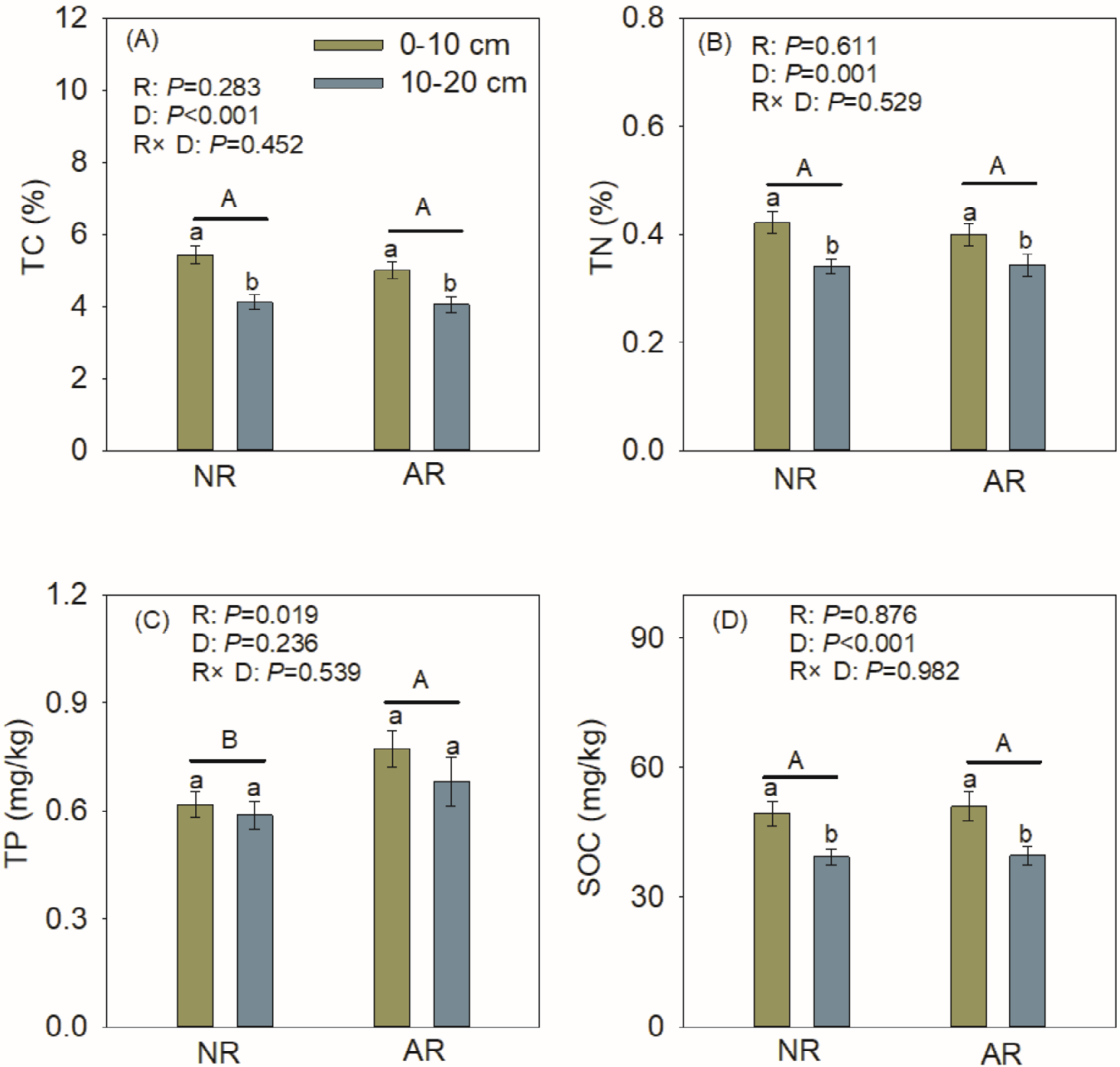
Effects of restoration methods and soil layer depth on soil nutrients. NR, AR, TC, TN, TP and SOC represent natural restoration, artificial restoration, soil total carbon, total nitrogen, total phosphorus and soil organic carbon respectively. Different uppercase letters indicate significant differences in the soil nutrients different restoration types (*p* < 0.05), while different lowercase letters indicate significant differences in the soil nutrients among different soil layer depths under the same restoration type (*p* < 0.05).

The soil total phosphorus content of artificially restored communities was significantly higher than that of naturally restored communities (Figure 6C), whereas no significant differences were detected in soil total nitrogen, total carbon, and organic carbon contents between the two restoration modes (Figure 6A,B,D). In contrast, the contents of soil total nitrogen, total carbon, and organic carbon in the 0–10 cm soil layer were significantly higher than those in the 10–20 cm soil layer (Figure 6A,B,D).

### 3.5 Responses of Soil Microbial Biomass and Enzyme Activity to Restoration Regimes and Soil Layer Depths

**FIGURE 7:**
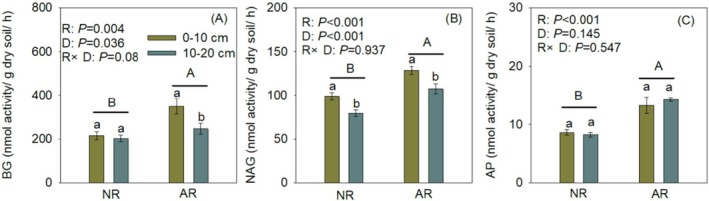
The effects of restoration methods and soil depth on soil microbial biomass and soil enzyme activity. BG, NAG and AP denote β‐glucosidase (BG), N‐acetyl‐β‐glucosidase, acid phosphatase (AP), respectively. Different uppercase letters indicate significant differences in the soil enzyme activity different restoration types (*p* < 0.05), while different lowercase letters indicate significant differences in the soil enzyme activity among different soil layer depths under the same restoration type (*p* < 0.05).

The contents of BG, N‐acetylglucosaminidase (NAG), and AP in the soil of artificially restored communities were all higher than those in naturally restored communities (Figure 7A–C); among them, the contents of BG and NAG in the 0–10 cm soil layer were significantly higher than those in the 10–20 cm soil layer (Figure 7A,B).

### 3.6 Correlations Among Soil Microbial Biomass, Soil Properties, and Plant Diversity

Soil microbial biomass carbon and microbial biomass nitrogen (MBN) were both significantly positively correlated with soil water content, but showed no significant correlations with soil pH, EC, and plant diversity (Table 1). In contrast, microbial biomass phosphorus was significantly positively correlated with soil EC, the Shannon Index, the Simpson Index, and the Pielou Index, whereas no significant correlations were observed between microbial biomass phosphorus and soil water content or pH (Table 1).

**TABLE 1:** Pearson's correlation coefficients among soil properties, soil microbial biomass, and plant diversity.

	SMC	pH	EC	MBC	MBN	MBP	Species Richness	Shannon Index	Simpson Index	Pielou Index
SMC	1	0.298	0.316	0.411*	0.477**	0.199	0.326	0.506**	0.444**	0.363*
pH	0.298	1	0.235	−0.084	−0.1	0.229	0.099	0.348*	0.377*	0.353*
EC	0.316	0.235	1	0.085	0.094	0.597**	−0.047	0.672**	0.761**	0.745**
MBC	0.411*	−0.084	0.085	1	0.767**	−0.082	−0.085	0.01	0.105	0.106
MBN	0.477**	−0.1	0.094	0.767**	1	−0.119	0.103	0.092	0.067	0.06
MBP	0.199	0.229	0.597**	−0.082	−0.119	1	−0.282	0.473**	0.670**	0.715**
Richness	0.326	0.099	−0.047	−0.085	0.103	−0.282	1	0.446**	0.07	−0.213
Shannon	0.506**	0.348*	0.672**	0.01	0.092	0.473**	0.446**	1	0.908**	0.770**
Simpson	0.444**	0.377*	0.761**	0.105	0.067	0.670**	0.07	0.908**	1	0.945**
Pielou	0.363*	0.353*	0.745**	0.106	0.06	0.715**	−0.213	0.770**	0.945**	1

*Note:* * and ** indicate significant correlations at the 0.05 and 0.01 levels (two‐tailed), respectively.

Abbreviations: EC, electrical conductivity; MBC, microbial biomass carbon; MBN, microbial biomass nitrogen; MBP, microbial biomass phosphorus; SMC, soil moisture content.

### 3.7 The Explanatory Power of Plant Diversity, Microbial Biomass, and Soil Properties With Respect to the Soil Multifunctionality Index

**FIGURE 8:**
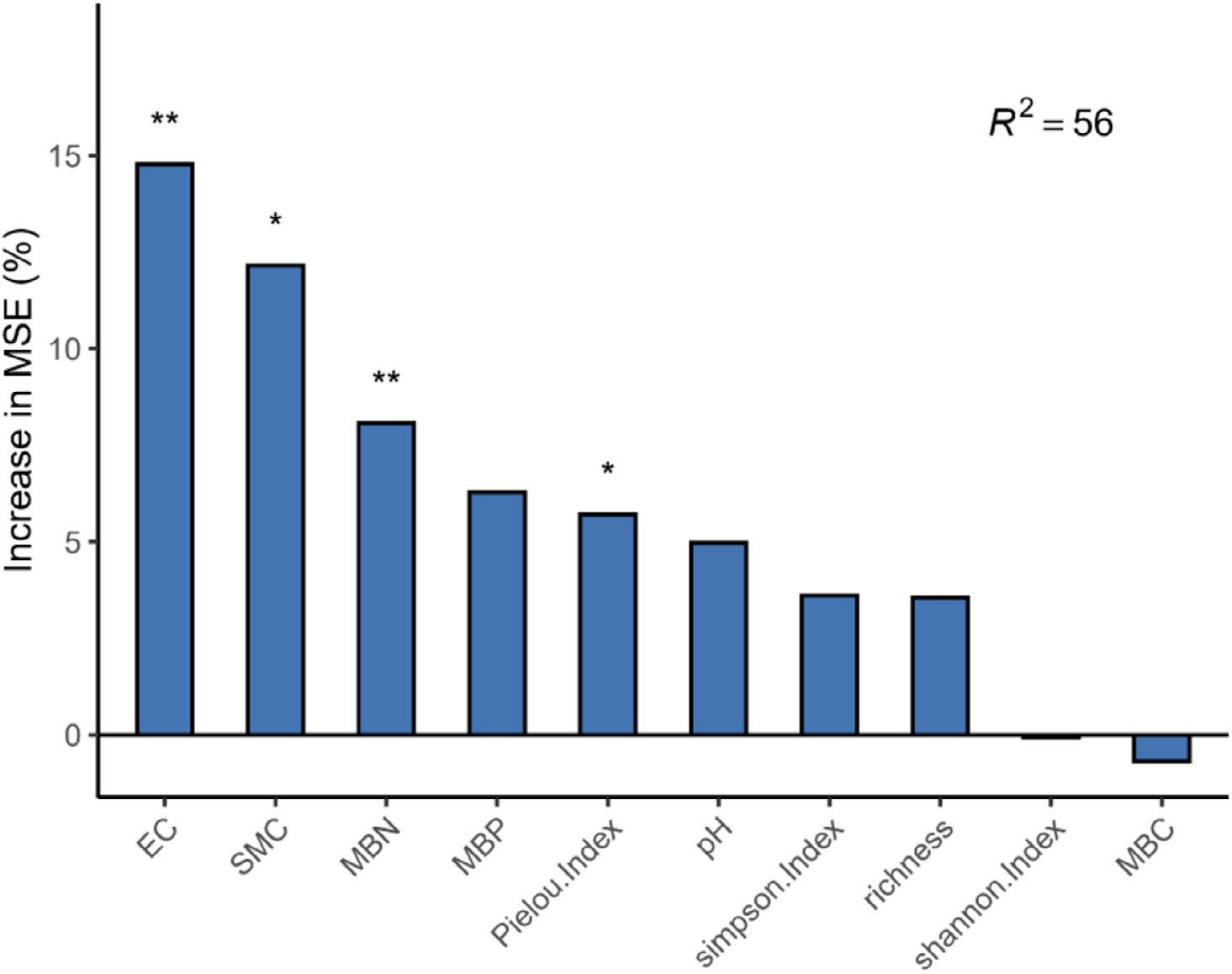
Explanation of the soil multifunctionality index by biotic factors and abiotic factors. *, **indicates that the interpretation of independent variable has a significant effect on the dependent variable (*p < 0.05, **p < 0.01). EC, electrical conductivity; SMC, soil moisture content; MBN, biomass carbon; MBP, microbial biomass nitrogen; MBP, microbial biomass phosphorus; MBC, microbial biomass phosphorus.

The model explains 56% of the variation (*R* ^2^ = 56). Among the variables, those contributing the most to the model error (MSE) are, in order, electrical onductivity (EC), soil moisture content (SMC), MBN and pielou index, all of which have a significant positive impact on soil multifunctionality (*p < 0.05, **p < 0.01) (Figure 8).

## 4 Discussion

### 4.1 Effects of Restoration Modes and Soil Depths on Soil Microbial Biomass

Soil microbes play a key role in biogeochemical cycling and ecosystem stability. Different restoration approaches affect soil microbial biomass by altering soil–vegetation–microbe relationships, and previous studies have found variations in microbial biomass between natural and artificial restoration due to differences in soil properties (Wang et al. 2024; Yang et al. 2020). In this study, no significant differences were observed in soil MBC and MBN between restoration types, but MBP was significantly higher under artificial restoration. Soil moisture is essential for microbial life activities: it not only maintains normal osmotic pressure of microbial cells and ensures metabolic enzyme activity but also promotes the dissolution of soil organic matter and diffusion of nutrients, providing favorable conditions for microbial synthesis of C and N containing substances. After 40 years of restoration, the overall soil moisture conditions under both restoration modes have stabilized and can meet the basic requirements for microbial C and N metabolism, which may explain the non‐significant differences in MBC and MBN between the two restoration types (Ramirez et al. 2012). Phosphorus in *Larix principis‐rupprechtii* needle litter is mainly in organic forms. With long‐term accumulation, microbes capable of organic P mineralization become enriched, and their secreted phosphatase efficiently breaks down organic P to release abundant available P (Qi et al. 2022). Pearson correlation analysis showed a positive relationship between soil available P and EC. This is because litter decomposition and root exudation release K, Ca, and other base cations, which together with activated P ions increase soil EC. Higher EC strengthens soil solution ionic strength, enhancing microbial uptake of P via ion exchange and thus promoting MBP accumulation. Naturally restored sites, by contrast, have lower base cations, available P, and EC, restricting MBP storage. This supports the significant positive correlation between MBP and EC (Sanz‐Luque et al. 2020). Furthermore, artificially restored communities feature multiple dominant plant species, providing diverse P activation pathways and substrates that support more P‐cycling microbial taxa. Microbial functional complementarity then improves P use efficiency and increases total MBP.

Soil depth is recognized as a pivotal abiotic factor mediating the vertical distribution pattern of soil microbial biomass, exerting a pronounced regulatory effect on its vertical stratification (Joergensen and Wichern 2018). Soil microbial biomass generally decreases with increasing soil depth. Topsoil contains abundant labile organic matter and favorable environmental conditions, supporting higher microbial activity and biomass. In deeper soil layers, organic matter inputs decline sharply, and poor aeration and permeability lead to lower microbial biomass. Concurrently, the availability of nutrients and the concentration of oxygen decrease progressively, which imposes stringent constraints on microbial growth and reproduction, thereby driving the gradient decline in soil microbial biomass across soil profiles (Blume et al. 2002; Van Leeuwen et al. 2017). Mirroring these general patterns, the findings of the present study demonstrated that soil microbial biomass carbon (MBC) and MBN in the 0–10 cm layer were significantly higher than those in the 10–20 cm layer under both restoration regimes. This observation aligns with prior results from diverse ecological settings. As an illustration, Lepcha and Devi (2020) documented greater soil MBC in the 0–15 cm versus 15–30 cm layer in the eastern Himalayas. Similarly, investigations on soil MBC and MBN dynamics in forest ecosystems of Northeast China revealed that, irrespective of stand types (natural secondary forests vs. *Larix gmelinii* plantations), the topsoil layers consistently harbored significantly higher MBC and MBN contents than the subsoil layers (Yang et al. 2010).

### 4.2 Effects of Restoration Regimes and Soil Depth on Soil Multifunctionality

Forest restoration regulates vegetation composition, litter input, and microbial community structure, thereby shaping soil multifunctionality. Studies on degraded forestlands in the red soil hilly region of southern China showed that 20‐year natural restoration plots had a soil organic matter content of 28.6 g/kg, with urease, phosphatase, and cellulase activities 42% higher than in *Pinus massoniana* plantations, indicating superior soil multifunctionality in naturally restored sites (Guo et al. 2024). Similarly, a global paired dataset revealed that naturally regenerated forests outperformed plantations in soil conservation, water retention, carbon sequestration, and biodiversity conservation (Hua et al. 2022). However, some studies found plantations have better soil functions than natural enclosures, such as a study in the karst rocky desertification area of northwestern Guangxi showing artificial restoration more effectively improved soil nutrients and enzyme activities (Lu et al. 2022). Consistent with our first hypothesis, soil multifunctionality differed between communities restored via artificial planting of *Larix principis‐rupprechtii* and naturally restored communities, with the former exhibiting higher soil multifunctionality. This can be attributed to the fact that artificially assisted communities are composed of multiple dominant species, and such multispecies communities enhance litter decomposition efficiency and soil nutrient availability through species complementarity effects. Mirroring the conclusions of Lu et al. (2022), these results confirm that artificially assisted restoration exerts a more pronounced positive effect on soil inorganic nitrogen content, soil enzyme activity, and the soil multifunctionality index than does natural restoration.

Our results also showed that soil multifunctionality was significantly higher in the topsoil (0–10 cm) than in the subsoil (10–20 cm), consistent with most previous studies. Plant litter and root exudates are mainly decomposed in the topsoil, directly supplying organic matter, available N, and available P. By contrast, nutrient accumulation in the subsoil is limited by the slow downward movement of decomposition products. Moreover, the topsoil provides better oxygen, temperature, and moisture conditions for microbial activity, whereas the subsoil has higher bulk density, lower porosity, and anaerobic conditions that suppress aerobic microbial metabolism and nutrient transformation (Rumpel and Kögel‐Knabner 2011; Singh et al. 2018).

### 4.3 Biotic and Abiotic Drivers of Soil Multifunctionality

The results of our random forest model verified the second hypothesis revealing that soil EC, soil moisture, MBN, and plant evenness index all exert significant impacts on soil multifunctionality. This can be explained by the following mechanisms: soil moisture directly drives the accumulation of available nutrients and the enhancement of enzyme activity by regulating nutrient dissolution, microbial metabolism, and enzyme structural stability (Bogati et al. 2025). In contrast, soil EC indirectly strengthens the functions of nutrients and enzymes by supplementing nutrient ions, regulating soil pH conditions, and activating enzymatic reactions (Pan et al. 2013). The synergistic interaction between these two abiotic factors constructs a stable soil physicochemical‐biological interaction system, which in turn maintains the soil's capacity for supplying available nutrients and its enzyme activity, ultimately enhancing soil multifunctionality. A large body of evidence has verified that biodiversity serves as a primary regulator of ecosystem functions. Although plant richness showed no significant regulatory effect on soil multifunctionality in our study, the plant evenness index proved to be a robust predictor of variations in soil multifunctionality. The artificially assisted restored communities exhibited a relatively high plant evenness index, with *Larix principis‐rupprechtii*, *Quercus wutaishanica*, and *Tilia* spp. accounting for considerable importance values. The divergent demands for light, water, and soil nutrients among these species enable sufficient resource complementarity (Zhao et al. 2024), thereby avoiding excessive consumption of specific resources by single dominant species such as *Quercus wutaishanica*. Moreover, plant communities with high evenness can input diverse organic matter into the soil: labile organic components provide readily available energy for soil microorganisms, while recalcitrant components contribute to the buildup of soil carbon pools and the optimization of soil structure, which further sustains the stability and activity of microbial communities (Li et al. 2024). In contrast, plant communities with low evenness tend to input a single type of organic matter, which may simplify the structure of microbial communities and constrain the soil nutrient cycling function. Extracellular enzymes, primarily secreted by soil microorganisms, play a crucial role in the decomposition of organic matter. Our findings demonstrated that MBN is a key determinant of soil multifunctionality. Consistent results have been reported in previous studies conducted in forest ecosystems (Delgado‐Baquerizo et al. 2016) and grassland ecosystems (Cui et al. 2020). This phenomenon can be attributed to the fact that different restoration approaches alter plant community structure, which in turn modulates the diversity of soil fungal and bacterial communities, regulates the rate of soil nutrient cycling, and thus affects soil multifunctionality (Roy et al. 2023).

## 5 Conclusion

This study compared natural and artificially assisted restoration over a 40‐year period in the Liupan Mountains and found that artificial restoration significantly improved soil multifunctionality, which also declined with increasing soil depth. Soil EC, water content, MBN, and plant evenness were identified as core drivers that regulate soil multifunctionality through soil–plant–microbe interactions. These findings underscore that artificial assistance effectively accelerates the recovery of ecosystem functions and provides a scientific foundation for the restoration of degraded temperate forests in China. However, this study did not assess the effects of different restoration methods on microbial diversity and soil animals diversity. In the future, we will further investigate changes across different trophic levels.

## Author Contributions

**Yonghong Luo:** funding acquisition (equal), investigation (equal), project administration (equal), writing – original draft (equal), writing – review and editing (equal). **Ye Liu:** formal analysis (equal). **Peng Wang:** writing – review and editing (equal). **Jiazhi Wang:** formal analysis (equal). **Fuji Liu:** writing – review and editing (equal). **Shuhua Wei:** data curation (equal). **Xingfu Yan:** conceptualization (equal), methodology (equal), supervision (equal). **Jiming Cheng:** funding acquisition (equal), investigation (equal), project administration (equal), writing – original draft (equal).

## Funding

This study was financially supported by the Natural Science Foundation of Ningxia Hui Autonomous Region (2026AAC050054, 2025AAC030050); Fundamental Research Funds for the Central Universities of North Minzu University, (2025QNPY11, 2025QNPY12, 2025XYZSK03); Ningxia Hui Autonomous Region Key R&D Program Project, (2026BEG02079); National Natural Science Foundation of China (32560296, 32560127); Youth Science and Technology Talent Support Project of Ningxia Hui Autonomous Region (2025).

## Ethics Statement

The authors have nothing to report.

## Conflicts of Interest

The authors declare no conflicts of interest.

## Supporting information


**Data S1:** ece373642‐sup‐0001‐DataS1.xls.

## Data Availability

All data generated or analyzed during this study are included in this published article and Data S1.
